# Knowledge, attitudes, and practices related to dengue among public school teachers in a Central Luzon Province in the Philippines: an analytic cross-sectional study

**DOI:** 10.1186/s41182-024-00591-7

**Published:** 2024-03-15

**Authors:** Ernesto R. Gregorio, Rie Takeuchi, Paul Michael R. Hernandez, John Robert Medina, Shin-ya Kawamura, Mikaela B. Salanguit, Marian Danille C. Santillan, Kimberly Mae S. Ramos, Gideon John Tuliao, Lyndon Morales, Maylin Palatino, Fumiko Shibuya, Jun Kobayashi

**Affiliations:** 1 Department of Health Promotion and Education, College of Public Health, University of the Philippines Manila, 625 Pedro Gil St, Ermita, 1000 Manila, Metro Manila Philippines; 2 SEAMEO-TROPMED Regional Center for Public Health, Hospital Administration, and Environmental and Occupational Health, Manila, Philippines; 3 Graduate School of Public Health, International University of Health and Welfare, 4‑3 Kodunomori, Narita, Chiba 286‑8686 Japan; 4 Department of Environmental and Occupational Health, College of Public Health, University of the Philippines Manila, Manila, Philippines; 5 National Institutes of Health, University of the Philippines Manila, Manila, Philippines; 6 Chubu Institute for Advanced Studies, 1200 Matsumoto‑Cho, Kasugai, Aichi 487‑8501 Japan; 7 College of Medicine, University of the Philippines Manila, Manila, Philippines; 8 Department of Education, Schools Division Office, City of San Fernando, Pampanga, Philippines; 9 School of Medicine, University of Maryland Baltimore, Baltimore, MD USA; 10 Department of Global Health, Graduate School of Health Sciences, University of the Ryukyus, Nishihara, Japan; 11 Japanese Consortium for Global School Health Research, University of the Ryukyus, Nishihara, Japan

**Keywords:** Dengue, Knowledge, Attitude, Practice, KAP

## Abstract

**Background:**

Dengue has become a major health issue in tropical regions as the numbers of reported cases and estimated infections continuously increase. In the Philippines, many challenges remain in preventing and controlling the disease amidst all the mitigation efforts of the government. This study sought to measure the health literacy of Filipino teachers and determine the associations among teachers’ knowledge, attitudes, and selected practices (KAP) against dengue.

**Methods:**

Elementary and secondary school teachers from the consistently declared dengue hotspots in the City of San Fernando, Pampanga, Philippines, from the years 2017 to 2019 were selected as target participants in this cross-sectional study. A self-administered online survey tool was used in this study for both screening of participants and the KAP survey proper. STATA, descriptive statistics, and multiple logistic regression were used for the data analysis. Odds Ratios (ORs) and 95% confidence intervals (CIs) were reported.

**Results:**

The study comprised 604 participants whose mean age was 38.4 years. Television was determined as the top media source of information, and various health staff were the most trusted and common source of information. Good knowledge on dengue treatment (OR = 1.81; 95% CI 1.18–2.78) and dengue prevention (OR = 1.85; 95% CI 1.26–2.71) were positively associated with having good practices on protection against mosquito bites. Good knowledge on dengue signs and symptoms (OR = 1.56; 95% CI 1.02–2.37) and dengue prevention (OR = 2.38; 95% CI 1.59–3.58) were positively associated with having good practices on preventing breeding sites. Those with positive perceived susceptibility to dengue had lower odds of having good practices on protection against mosquito bites (OR = 0.64; 95% CI 0.41–0.99) and of having good practices on preventing breeding sites (OR = 0.46; 95% CI 0.26–0.81).

**Conclusion:**

Even with the existing dengue policies, programs, and strategies, and the high disease literacy rate of Filipinos, dengue remains a struggle with an increasing case rate. Therefore, specific concepts should be emphasized, and interventions should be fine-tuned to better reach and influence the target population to attain a dengue-free Philippines.

## Introduction

The majority of low- and middle-income countries are in the tropics where the spread of neglected tropical diseases has become a major public health crisis. Dengue, in particular, has become a global threat due to the increasing number of infected people and endemic areas, which is strongly influenced by global warming. According to the Philippines’ Department of Health, dengue is the fastest-spreading vector-borne disease in the world, being endemic in 100 countries [1]. In Southeast Asia, the number of reported dengue fever outbreaks has increased dramatically [2]. About 100 million cases of dengue occur each year along with an estimated 400 million infections. Forty percent of the world’s population, or about 3 million people, live in areas with risk of the disease [2–4].

Due to the limited resources in low- and middle-income countries, correct diagnosis of dengue has become even more important. Early detection of the disease leads to administration of timely and appropriate medical interventions. This has lowered the fatality rate of severe dengue from 20% to below 1% [2]. However, the diagnosis of dengue has proved to be challenging due to its nonspecific presentation, especially during its early stages [5]. With the limited resources available, low accessibility to healthcare, and hesitancy to go to hospitals, compounded by the challenges brought about by the COVID-19 pandemic, differential diagnosis of febrile diseases is expected to be very difficult [6]. For example, during the early phase of the COVID-19 pandemic, dengue and COVID-19 were incorrectly diagnosed as both patients presented with lymphopenia, leukopenia, thrombocytopenia, and elevated transaminases in laboratory tests. In addition, false information that COVID-19 is transmitted by mosquitoes has become a problem in low- and middle-income tropical countries where mosquito-borne diseases such as dengue are widespread. While this could be protective to a certain extent, the said misinformation still poses a challenge in terms of the diagnosis and treatment of dengue. Therefore, risk communication to convey appropriate information is important in febrile disease control.

Challenges remain in controlling the spread of dengue despite the presence of Philippine government’s prevention policies and an established case reporting system. Contributing to the Philippines’ situation of increased dengue cases is the 2017 dengue vaccine controversy involving Dengvaxia. Yu et al. discovered that after the controversy, there is widespread mistrust and fear in various communities towards the state and health authorities [7]. The researchers also added that the media played a role in the public's vaccine hesitancy, as well as the altered perceptions towards the government and healthcare. Therefore, it is essential to efficiently implement prevention programs through vector control strategies, appropriate health behavior change, and early diagnosis and appropriate treatment.

Although dengue fever affects all age groups, it is still most common among older children, adolescents and adults [8]. In a Knowledge, Attitude, and Practice (KAP) study conducted on primary school children in Thailand, it was discovered that the main sources of information on dengue were primary school teachers and guardians and that most of the study participants possessed poor dengue-related knowledge [9]. The health literacy of school teachers related to dengue must be assessed to see how well they respond to cues important for school-based vector surveillance. Health literacy may then be further enhanced to improve their surveillance responses through appropriate school health education, with a focus on febrile diseases important in the public health context.

In 2022, the Philippine government began to lift the COVID-19 pandemic restrictions, and dengue was expected to become even more widespread. As the number of cases continues to increase, it is important to understand these implications and take appropriate measures. With the resumption of face-to-face classes in the Philippines, health literacy in schools can be an important area for intervention in the Philippines where underreporting of dengue fever cases exists [10]. Misinformation such as “COVID-19 is transmitted by mosquitoes” should be corrected. To further amplify the current surveillance, prevention, and control measures of dengue cases in the Philippines, knowledge of the factors that affect the rise of dengue cases is vital in implementing appropriate measures.

A study conducted in Lao People’s Democratic Republic found that knowledge about climate change and dengue was significantly associated with one’s level of education and socioeconomic status [11]. In addition, attitudes toward climate change and dengue were associated with internet use and level of education whereas practices related to dengue were associated with internet use, level of education, socioeconomic status, and previous dengue experience. From this study, it can be seen that one’s knowledge, attitudes, and practices toward dengue are highly dependent on their level of education. In the Philippine context, Labrague and Yboa conducted a study among rural residents in Samar, Philippines [12]. Despite access to correct information on dengue, the disease remains as a challenge. They found that there are high levels of knowledge and preventive practices regarding dengue. However, higher levels of knowledge do not necessarily lead to better practices of prevention. With this information, it is also important to determine the associations among knowledge, attitudes, and practices to determine which aspects may be the focus of health communication program that can lead to better health outcomes.

This study thus targeted the health literacy of Filipino teachers who serve as one of the sources of health information about dengue. The study aimed to determine the association between the knowledge, attitudes, and selected practices of teachers on dengue and specifically to (1) determine the respondents’ knowledge on the modes of transmission, signs and symptoms, diagnosis, treatment, and methods of preventing dengue; (2) assess the respondents’ attitudes towards dengue prevention and control measures; (3) determine the proportion of respondents who practice health-seeking behaviors and prevention and control measures against dengue; (4) determine the association between the respondents’ prevention and control practices, and their knowledge and attitudes towards dengue and COVID-19; and (5) determine the primary sources of information of the respondents on dengue.

## Methods

### Research design

An analytical cross-sectional design was used to assess the knowledge, attitudes, and practices of public elementary and junior high school teachers regarding dengue. The data were collected from September 2022 to November 2022.

### Study site

The City of San Fernando, Pampanga, in the Philippines was chosen purposively as the study site. The researchers obtained data on the dengue cases from 2017 to 2019 in the City of San Fernando, Pampanga from their City Health Office  in coordination with their Department of Education Schools Division Office (SDO) to identify the villages (barangays) that had consistently been dengue hotspots, that is, areas with confirmed clustered cases that had increased in number during the past four weeks [13]. Based on this information, nine public elementary schools and 10 junior high schools in the identified villages were selected.

### Study population

The target participants of the study were public elementary and junior high school teachers of the villages that have consistently been dengue hotspots in the City of San Fernando, Pampanga. This study included all eligible teachers in the selected schools. The inclusion criteria were (a) currently employed or on active duty for the school year 2021–2022; (b) teaching staff; and (c) age 18 to 64 years old. A questionnaire that included consent to participation and data inclusion in the study was used for the initial screening. Participants were excluded from the study if they did not provide consent.

### Research instrument

The KAP survey instrument was developed based on existing literature and survey tools including a similar study on Zika Virus, which referenced an instrument provided by the World Health Organization (WHO)-Pan American Health Organization [14].

The self-administered tool consisted of six domains. The first domain contained eight questions that collected the participants’ sociodemographic characteristics. The second domain had four questions relating to any history of exposure to dengue. The third domain had 20 questions about the participant’s knowledge on the dengue virus, which was further categorized into mode of transmission, signs and symptoms, diagnosis, treatment, and prevention. Mode of transmission refers to the participants’ knowledge on how dengue virus is spread from one susceptible host to another, which was measured using seven questions. Signs and symptoms refer to the participants' knowledge of the clinical signs and symptoms of dengue and was measured using two questions. Diagnosis refers to the participants’ knowledge of the correct method of dengue diagnosis, which was measured using five questions. Treatment refers to the participants' knowledge of appropriate treatment for dengue and was measured using four questions. Lastly, prevention refers to the participants' knowledge on how dengue can be prevented. This was measured using two questions. The transmission, diagnosis, and treatment categories in the knowledge domain were measured using true or false statements, with the option to choose “I don’t know.” Meanwhile, the signs and symptoms and prevention categories used multiple-answer questions.

The fourth domain had 15 items that measured the attitudes of the participants towards dengue control, prevention, and treatment. For this, the study utilized the constructs of the health belief model, specifically, perceived benefit, perceived barriers, perceived susceptibility, perceived severity, and external cues to action. All constructs were measured using three questions that were answered through a modified Likert Scale. Perceived benefit refers to the participants' belief in the effectiveness of proper actions to reduce the risks related to dengue. Perceived barriers refer to the participants' belief that tangible and intangible barriers such as costs and safety concerns can prevent the enactment of the advised action. Perceived susceptibility refers to the belief of the participants about their chances of contracting dengue. Perceived severity refers to the belief of the participants about the serious clinical effects of dengue. External cues to action refer to the people or personalities that may possibly influence the participant’s decision to enact the advised action.

The fifth domain contained five questions, four of which could have multiple answers regarding the sources of information and health literacy. The sixth domain measured the participants' practices on dengue prevention and health-seeking behaviors through an item that can have multiple answers. Five questions answered with a four-point Likert scale on their perceptions on the COVID-19 pandemic’s effect on the government’s response in fighting dengue and an open-ended question on the most appropriate strategy for teaching dengue prevention were included in this domain.

To test the reliability of the survey tool, Kuder–Richardson 20 and Cronbach’s Alpha were used with result interpretation considered to be highly reliable.

### Data collection, management, and analysis

A letter of request to conduct the study was sent to the SDO Superintendent and to the school heads. Once permission was obtained, assistance from the School Governance and Operations Division was sought to enable the research team to gain access to the teachers. The link to the study questionnaire was sent through an email to the school head of each of the selected schools and was then distributed to the teachers through their group chats. A printed QR code for the questionnaire was also distributed to help facilitate the dissemination of the survey to the teachers. The settings of the online tool used for the study questionnaire ensured that all fields and questions were answered before submission. If the submitted answer was not applicable to the question, the cell was then left blank and was not included in the logistic regression analysis. All individual questionnaire responses were double-checked and verified for completeness and consistency before processing.

The data collected were encoded through Microsoft Excel (Microsoft Corporation, Redmond, WA, USA). Correct answers in the knowledge domain items were coded as 1, 0 otherwise. The percentages of correct answers of the participants for each knowledge domain were computed. A score of more than 75% was considered as having good knowledge, coded as 1, poor knowledge otherwise, coded as 0. For the attitude domain, the responses of 4 and 3 to the Likert scale were coded as being answers with favorable (positive) attitudes towards dengue prevention and control, whereas 2 and 1 were answers indicating less favorable (negative) attitudes towards dengue prevention and control.

STATA 16.1 (STATA Corp., College Station, TX, USA) and descriptive statistics were used for data analysis of the survey, including the sociodemographic profile and knowledge, attitudes, and practices of the participants. For continuous variables such as age of the participants, means and standard deviations were calculated while frequencies and percentages were computed for categorical variables.

Two binary prevention and protection practice outcome variables were defined, namely, (1) practices on protection from mosquito bites; and (2) practices on preventing breeding sites, which included four and five items, respectively. For each outcome, good practice was defined as doing at least 75% of the included items. Binary logistic regression modeling was employed to determine the factors in the knowledge and attitude domains that were associated with the outcome variables, while controlling for confounding of age, sex, educational attainment and subject taught.

## Results

### Sociodemographic profile

Of the 987 teachers, 618 answered the questionnaire, giving a response rate of 62.6%. Of the 606 teachers who agreed to participate, 604 were eligible for the study and analysis, the two participants who were not part of the teaching staff were excluded from the study. Thus, 604 respondents participated in the study. Their average age was 38.4 (S.D. 10.2; range: 20–62) years. The majority were females (81.6%), had a master’s level or were a master’s graduate (58.8%), and had a family income between PHP 25,200 and PHP 95,299 monthly. About a quarter reported teaching Science or Music, Arts, Physical Education, and Health (Table 1).

**Table 1:** Characteristics of the participating public elementary and secondary school teachers, Pampanga, Philippines (*n* = 604)

Characteristic	Frequency (%)
Age (in years)	
Mean (S.D.)	38.4 (10.2)
20–29	155 (25.6)
30–39	186 (30.7)
40–49	161 (26.6)
50–59	90 (14.9)
60 and above	11 (1.8)
No response	1 (0.2)
Sex	
Male	101 (16.7)
Female	493 (81.6)
Prefer not to say	10 (1.7)
Civil status	
Single	190 (31.5)
Married	393 (65.1)
Separated/widowed	21 (3.0)
Educational attainment	
College level/graduate	234 (38.7)
Master level/graduate	355 (58.8)
PhD level/graduate	15 (2.5)
Monthly family income	
< 25,200	142 (23.5)
25,200—95,299	458 (75.8)
> 95,299	4 (0.7)
Subject taught	
Science/MAPEH	147 (24.3)
Other	457 (75.7)

More than half (65.1%) of the respondents were married individuals. Three-quarters (75.8%) declared themselves to be in the middle-income bracket.

Almost half (48.7%) of the participants were currently taking up their master’s degree, while 34.4% were college graduates. Almost a quarter (24.3%) of the participants taught Science and Music, Arts, Physical Education, and Health.

### Sources of information on dengue

The respondents’ commonly used media platforms on obtaining dengue information were television (85.3%), Facebook (79.6%), and Youtube (52.3%), whereas the social media app Viber (3%) and comics (2.7%) were the least preferred.

The respondents’ top three primary sources of data on dengue were Department of Health (DOH) officials (83%), Rural Health Unit staff (64.4%), and members of the Barangay Health Emergency Response Team (56.1%). Other sources (4.1%) and the Department of Education officials (29.8%) were ranked the lowest.

DOH officials (89.1%), Rural Health Unit staff (63.6%), and WHO officials (58.4%) were the top three most trusted sources of information about dengue. The most trusted source of information among non-health officials/employees was family members (34.6%).

### Knowledge on dengue

Table 2 shows that the majority of the respondents  knew that skin-to-skin contact (95.5%) and sexual intercourse (90.7%) cannot transmit the disease. Only over half of the respondents (53.5%) knew that dengue can be transmitted through blood transfusion, which is low compared to the other items.

**Table 2:** Frequency and percent distribution of respondents according to knowledge on dengue, Philippines, 2022 (n = 604)

Knowledge item	Frequency of correct response (%)
Knowledge on the modes of transmission	
Skin-to-skin contact cannot transmit dengue	577 (95.5)
Dengue cannot be transmitted through sexual intercourse	548 (90.7)
Not all types/species of mosquitoes can transmit the dengue virus	478 (79.1)
Dengue mosquitoes bite humans during daytime	450 (74.5)
COVID-19 cannot be transmitted via a mosquito bite	443 (73.3)
Dengue can be transmitted through blood transfusion	323 (53.5)
Knowledge on signs and symptoms*	
Fever	584 (96.7)
Rashes	538 (89.1)
Headache	502 (83.1)
Muscle pain	457 (75.7)
Bleeding	428 (70.9)
Abdominal pain	328 (54.3)
Diarrhea	328 (54.3)
Eye pain	190 (31.5)
Knowledge on prevention and control*	
Removal of standing water at home and school	590 (97.7)
Use window screens and bed nets to prevent mosquito bites	567 (93.9)
Covering water containers	567 (93.9)
Using mosquito repellent as a self-protection measure	567 (93.9)
Fogging/spraying during a dengue outbreak	541 (89.6)
Seeking early medical consultation	494 (81.8)
Getting my child vaccinated against dengue	339 (56.1)
Getting myself vaccinated against COVID-19 is irrelevant	159 (26.3)
Knowledge on the diagnosis and treatment	
The diagnosis of dengue is based on blood samples	577 (95.5)
Only a physician can give the final diagnosis of dengue	564 (93.4)
Blood transfusion in treating dengue is beneficial to the patient	524 (86.8)
Some medicinal plants like Tawa tawa are effective in treating dengue	399 (66.1)
A positive result in dengue NS1 (rapid diagnostic test/RDT) suggests the diagnosis of dengue	395 (65.4)
Having a fever for one to seven days without any other symptoms cannot confirm the presence of dengue	341 (56.5)
If I have fever, I might have either dengue or COVID-19	295 (48.8)
There is currently no medicine which can cure dengue	264 (43.7)
Supportive treatment (e.g., staying well hydrated, drinking paracetamol and having sponge baths to treat fever) is advised for individuals with dengue	244 (40.4)
Diagnosis of dengue can be confirmed through a polymerase chain reaction (PCR) test	149 (24.7)

^*^Multiple responses are possible

More than half of the participants claimed that abdominal pain, bleeding, diarrhea, headache, and muscle pain are signs and symptoms of dengue, with fever (96.7%) and rashes (89.1%) as the most recognized symptoms linked to dengue (Table 2).

Respondents were assessed on their knowledge of preventive measures against dengue, which were divided into (1) prevention and control of the disease; and (2) diagnosis and treatment options. Table 2 shows that the majority of the respondents (> 80%) were able to correctly identify prevention and control methods for dengue, which are the following: removal of standing water at home and school, use of window screens and bed nets to prevent mosquito bites, covering water containers, using mosquito repellent as a self-protection measure, fogging/spraying during a dengue outbreak, with child vaccination identified by the lowest number of participants (56.1%).

Respondents agreed that the diagnosis of dengue is based on blood samples (95.5%), and only a physician can give the final diagnosis of dengue (93.4%), whereas more than half of them (75.3%) either disagreed on the diagnosis of dengue through a PCR test or did not know the answer (Table 2).

### Attitudes on dengue

Respondents were assessed on their perceptions of dengue, and the responses were categorized into (1) perceived benefits; (2) perceived barriers; (3) perceived severity; (4) perceived susceptibility; and (5) cues to action. Table 3 shows that respondents viewed the use of mosquito coils (62.7%) and application of mosquito repellants (78.5%) as beneficial in the prevention and protection against dengue.

**Table 3:** Perceptions of dengue of the respondents, Pampanga, Philippines, 2022 (*n* = 604)

Perceptions	Frequency of positive attitude (%)
Perceived benefits	
Wearing clothes that cover most parts of my body should be able to protect me from getting dengue	425 (70.4)
Using mosquito coils will lessen my chances of getting dengue	379 (62.7)
Applying mosquito repellant will protect me from getting dengue	474 (78.5)
Perceived barriers	
If the materials that can be used to protect myself against dengue are expensive, I will not buy these	393 (65.1)
If the health facilities are not accessible, I will not go to these facilities	339 (56.1)
If information regarding dengue prevention is not made available to me, I will not search it	494 (81.8)
Perceived severity	
I believe that dengue can lead to the more severe dengue hemorrhagic fever	594 (98.3)
I believe that there is no medicine currently available that can cure dengue	329 (54.5)
I believe that currently there are no available effective and safe vaccines against dengue	370 (61.3)
Perceived susceptibility	
I feel that I am at risk of contracting dengue in the next three months	405 (67.1)
I feel that my chances of getting dengue is high if the people around me have dengue	417 (69.0)
Presence of mosquito breeding sites increases my risk of getting dengue	589 (97.5)
Cues to action	
I should seek medical attention if I experience flu-like symptoms	596 (98.7)
I will protect myself against dengue if my family members or relatives recommend it	540 (89.4)
I will protect myself against dengue if my doctors recommend it	541 (89.6)

More than half of the respondents in Table 3 agreed that even if the materials that can be used to protect oneself against dengue are expensive (65.1%), health facilities are not accessible (56.1%), and information regarding dengue prevention is not made available (81.8%), they are still willing to avail of these options.

Almost all (98.3%) of the respondents in Table 3 either agreed or strongly agreed that dengue can lead to more severe dengue hemorrhagic fever, and more than half agreed that there is no currently available medicine (54.5%) or effective and safe vaccines (61.3%) against dengue.

Over half (67.1%) of the participants agreed that they feel they are at risk of contracting dengue in the next three months, whereas 69.0% and 97.5% feel that they are more susceptible when the people around them have dengue fever and if there are mosquito breeding sites in their area, respectively (Table 3).

Almost all (98.7%) of the respondents feel the need to seek medical attention when they experience flu-like symptoms. They will also protect themselves against the disease when family members or relatives (89.4%) or their doctors (89.6%) recommend it (Table 3).

### Practices related to dengue prevention

As shown in Table 4, the most commonly practiced preventive option for dengue done by the respondents in the past three months was the disposal of garbage or trash (88.1%). Although the use of mosquito coils was ranked the lowest, it is still being practiced by more than half of the respondents (57.8%).

**Table 4:** Dengue preventive practices done in the past three months by respondents, Pampanga, Philippines, 2022 (*n* = 604)

Prevention practices	Frequency (%)
Disposed of garbage/trash	532 (88.1)
Drained water containers used for plants	499 (82.6)
Drained water filled containers	487 (80.6)
Used screen windows	481 (79.6)
Emptied water containers around the house	455 (75.3)
Covered all water containers	454 (75.2)
Used insecticide sprays	442 (73.2)
Used a mosquito repellant	443 (73.3)
Used mosquito coils	349 (57.8)
Others	3 (0.5)

### Factors associated with dengue-related practices

For this part of the study, a total of 602 participants with complete data were included in the analyses.

#### Correlates of good practices on protection from mosquito bites

Controlling for age, sex, educational attainment, subject taught, and the other variables, those with good knowledge on the treatment of dengue had 1.81 times the odds of having good practices on protection from mosquito bites compared to those who had poor knowledge (aOR = 1.81; 95% CI 1.18, 2.78). Those with good knowledge on the prevention of dengue had 1.85 times the odds of having good practices on protection from mosquito bites compared to those who had poor knowledge (aOR = 1.85; 95% CI 1.26, 2.71). Those with positive perceived susceptibility to dengue had 36% lower odds of having good practices on preventing mosquito bites than those with negative perceived susceptibility (aOR = 0.64; 95% CI 0.41, 0.99) (Table 5).

**Table 5:** Factors associated with good dengue-related practices on protection from mosquito bites and preventing breeding sites (*n* = 602)

Factor	Practices on protection from mosquito bites		Practices on preventing breeding sites	
	Number with good practice per exposure category (% per category)	Adjusted OR (95% CI)	Number with good practice per exposure category (% per category)	Adjusted OR (95% CI)
Age in years		0.98 (0.96, 0.99)**		1.00 (0.98, 1.02)
Sex				
Female	189 (38.5)	1.00	381 (77.6)	1.00
Male	34 (33.7)	0.83 (0.52, 1.33)	58 (57.4)	0.42 (0.26, 0.68)
Prefer not to say	5 (50.0)	1.50 (0.39, 5.74)	6 (60.0)	0.61 (0.15, 2.60)
Educational attainment				
College level/graduate	91 (39.1)	1.00	169 (72.5)	1.00
At least master level	137 (37.1)	0.95 (0.66, 1.35)	276 (74.8)	1.20 (0.80, 1.80)
Subject taught				
Other	168 (36.8)	1.00	333 (73.0)	1.00
Science/MAPEH	60 (41.1)	1.17 (0.78, 1.74)	112 (76.7)	1.27 (0.79, 2.03)
Knowledge				
Modes of transmission				
Poor	121 (38.9)	1.00	230 (74.0)	1.00
Good	107 (36.8)	0.93 (0.66, 1.32)	215 (73.9)	0.90 (0.61, 1.34)
Signs and symptoms				
Poor	122 (35.7)	1.00	235 (68.7)	1.00
Good	106 (40.8)	1.07 (0.75, 1.53)	210 (80.8)	1.56 (1.02, 2.37)*
Diagnosis of dengue				
Poor	135 (38.4)	1.00	251 (71.3)	1.00
Good	93 (37.2)	0.87 (0.61, 1.25)	194 (77.6)	1.21 (0.80, 1.82)
Treatment of dengue				
Poor	171 (35.0)	1.00	355 (72.8)	1.00
Good	57 (50.0)	1.81 (1.18, 2.78)^**^	90 (79.0)	1.22 (0.72, 2.07)
Prevention of dengue				
Poor	61 (29.5)	1.00	129 (62.3)	1.00
Good	167 (42.3)	1.85 (1.26, 2.71)^**^	316 (80.0)	2.38 (1.59, 3.58)^↓^
Attitude				
Perceived barriers				
Negative	59 (36.0)	1.00	109 (66.5)	1.00
Positive	169 (38.6)	1.21 (0.82, 1.80)	336 (76.7)	1.58 (1.03, 2.44)^*^
Perceived severity				
Negative	38 (35.5)	1.00	76 (71.0)	1.00
Positive	190 (38.4)	1.00 (0.64, 1.57)	369 (74.6)	1.13 (0.70, 1.85)
Perceived susceptibility				
Negative	51 (45.1)	1.00	94 (83.2)	1.00
Positive	177 (36.2)	0.64 (0.41, 0.99)*	351 (71.8)	0.46 (0.26, 0.81)^**^
Internal cues to action				
Negative	2 (25.0)	1.00	5 (62.5)	1.00
Positive	226 (38.1)	2.06 (0.40, 10.74)	440 (74.1)	1.96 (0.41, 9.45)
External cues to action				
Negative	26 (32.9)	1.00	66 (83.5)	1.00
Positive	202 (38.6)	1.13 (0.66, 1.91)	379 (72.5)	0.53 (0.27, 1.02)

OR, odds ratio; CI, confidence interval; MAPEH: Music, Arts, Physical Education, and Health

^*^p-value < 0.05, **p-value < 0.010, ^↓^*p*-value < 0.001

#### Correlates of good practices on preventing breeding sites

Controlling for age, sex, educational attainment, subject taught, and the other variables, those with good knowledge on signs and symptoms of dengue and on prevention of dengue had 1.56 (95% CI 1.02, 2.37) and 2.38 (95% CI 1.59, 3.58) times the odds of having good practices on preventing breeding sites, respectively, compared to those with poor knowledge. Those with positive perceived barriers of using personal protective/preventive measures against dengue had 1.58 times the odds of having good practices on preventing breeding sites compared to those with negative attitudes (aOR = 1.58; 95% CI 1.03, 2.44). Finally, those with positive perceived susceptibility to dengue had 54% lower odds of having good practices on preventing breeding sites than those with negative perceived susceptibility (aOR = 0.46; 95% CI 0.26, 0.81) (Table 5).

## Discussion

This study evaluated the knowledge, attitudes, and preventive practices regarding dengue among public elementary and secondary school teachers in the City of San Fernando, Pampanga, Philippines. The participants showed good overall knowledge on the modes of dengue transmission, signs and symptoms, prevention and control, and diagnosis and treatment as more than half of the respondents were able to correctly answer most of the items. This could be attributed to the programs and strategies implemented by the Department of Health and the Local Government Unit of Pampanga to achieve its vision of a dengue-free Philippines [1]. This study result is also consistent with previous investigations reflecting high dengue knowledge levels in Filipino populations [12, 15]. However, even with the reported number of individuals knowledgeable about dengue, this study still shows that a certain proportion of the respondents either hold incorrect concepts or are unfamiliar with some facts about the disease.

Only about half of the participants could correctly identify that dengue can be transmitted through blood transfusion. Available information about transfusion-transmitted dengue is limited and could be the reason why this type of transmission is not a common knowledge among the respondents. Relatively, vaccination against dengue scored the lowest among the correct prevention and control measures, which could be attributed to the apprehensions toward vaccines as a result of the former Dengvaxia controversy. Official statistics conveyed the significant increase in vaccine hesitancy supported by individual reports on how immunization coverage rates dropped in the aftermath of the controversy [7]. On the contrary, the majority of the participants mistakenly believe that getting vaccinated against COVID-19 could lead to protection against dengue. Whether this identifies what the public considers as an alternative to obtaining protection against dengue by mode of vaccination merits further investigation. However, this also points to a significant concern about health communication to the public. In addition, only a low number of the participants correctly believe that a dengue diagnosis can be confirmed through PCR, that there is currently no medicine to cure dengue, and that having a fever for one to seven days without any other symptoms cannot confirm the presence of dengue. To bridge these gaps, emphasis on points regarding modes of dengue transmission, prevention, control, diagnosis, and treatment should be added to the different dengue awareness programs in the province. Specifically, the dengue awareness programs should focus on correcting the misconceptions surrounding vaccination to address vaccine hesitancy.

This study also reports an overall positive attitude towards dengue prevention and control measures, which is consistent on what was found in other Asian countries, although intervention studies have been suggested as points for improvement [16–18]. This shows that the majority of the respondents have positive perceptions of benefits and barriers to using personal dengue protection and are willing to undertake additional efforts for health safety. In addition, perceived severity and susceptibility show the participants’ awareness of the trends and risks of the disease. Internal and external cues to action against dengue was also found among the majority of the respondents. All of these perceptions could possibly be among the effects of various news reports and articles regarding the continuous and alarming increase of dengue cases from the start of the first half of the year 2022 until the start of the second half in the Philippines [19, 20].

A high proportion of respondents also claimed to have practiced, within the past three months, every health-seeking behavior, prevention, and control measures against dengue listed in this study. This could be attributed to the desire to remain vigilant against the disease despite the decrease in reported cases in Central Luzon, Philippines for the first four months of 2022 compared with the records reported for the same period in 2021 [21]. A similar practice result was seen in KAP investigations regarding dengue among Malaysian populations [22, 23]. In contrast, preventive practices against the disease in Singapore for the same year 2022 were reported to be low and could be attributed to the seemingly ineffective prevention regimens imposed on residents, thereby resulting in a reluctance to engage with dengue volunteers and national health officers [18].

This study likewise found that good knowledge on the treatment and prevention of dengue was associated with good practice of mosquito bite prevention. Moreover, good knowledge on the signs, symptoms, and prevention of dengue were associated with an increased odds of good practice of preventing breeding sites. These findings, however, were not consistent with the results reported from a study in Cebu City and Metro Manila Philippines where the knowledge of participants about dengue fever did not correlate with their practices against the disease [15, 24]. Differences in the ways of living between Pampanga, Metro Manila, and Cebu City could be a reason for this discrepancy, with a high dependency on paid workers doing the cleaning and other utility jobs in the latter two areas than the former area.

Finally, a positive association was observed between the respondents’ attitudes and practices on preventing breeding sites, such that a positive perception of barriers increases the likelihood of engaging in good practices. However, participants with a favorable attitude towards the perceived susceptibility to dengue, such as those who feel that they are at risk of contracting dengue, were less likely to practice prevention of dengue breeding sites compared to those with less favorable attitudes. This is in contrast with studies from Central Nepal and Indonesia where strong associations between a good attitude and good preventive practices were reported [16, 25]. A possible explanation for this is that while the perceived susceptibility has affected the participants’ intentions, but they did not have the opportunity to implement these practices. Therefore, appropriate programs should be developed to further translate attitudes into practice, such as through consistent garbage collection schedules and regular clean-up drives.

DOH officials, Rural Health Unit staff, barangay health emergency response teams, family members, and WHO officials are the top five primary and most trusted sources of dengue information for the study respondents. From this list, only family members are included among the non-health officials or employees. Empowering these specific people could serve great advantage in increasing good practices, given the reported high associations of good knowledge and preventive actions. In terms of media coverage, television and Facebook are the top sources of dengue information and could be used as means to better reach this particular segment of the population.

While significant statistical associations were observed in some variables, cause and effect relationship was not established as this is one of the limitations of the cross-sectional nature of this study. Moreover, the study results may not be generalized to the study population since a nonrandom sampling method was utilized in the selection of schools.

Second, the online means of survey distribution eliminated interviewer bias that could possibly occur when conducted using face-to-face interviews. However, the use of the digital platform might have introduced information bias as validation of each input from the respondents was difficult. Recall bias could have also occurred as one section of the survey involved remembering the practices done in the past three months and identifying any disease history for self, family, friends, and colleagues, with the latter event as a possible influence on knowledge about dengue. However, a previous study reported no association between having a family member with a history of dengue and obtaining increased knowledge on the disease [25].

Finally, this study assured the participants of the study’s confidentiality and that their identity will be protected in the survey. This might have reduced the information bias because they knew that their responses will not be traced back to them. This study’s internal validity was strengthened by controlling the effects of the confounding variables during the data analysis. As for the external validity, although the strict inclusion criteria could have limited the generalizability of the findings, a good participation and representation was achieved through a reminder from the SDO superintendents and school heads to ensure a timely and complete response from all of the eligible participants.

## Conclusion

Dengue is among the seasonal struggles in a tropical country like the Philippines. Even with its decades-worth of efforts and the high degree of dengue literacy of its citizens, the Philippines still struggles in combating the disease, especially with the emergence of additional challenges influencing the knowledge, attitude, and practices of Filipinos. The findings of this study can serve as baseline health literacy of teachers in the province on dengue, and can be used as a guide in the review and redesign of school-based dengue prevention programs and strategies to fit the status and needs of the teachers who can influence the learners and, to a certain extent, the general population. The in-service training programs for Filipino teachers can include topics on dengue with emphasis on the findings where teachers have low level of knowledge. Based on the findings from this study, learning modules for teachers as well as students can be developed to improve their dengue literacy. Moreover, the significant factors associated with dengue prevention and control derived from this investigation could be used in developing health communication materials with messages that can influence the target population in the province and thereby contributing to the attainment of the vision of a dengue-free Philippines.

## Data Availability

The datasets generated and/or analyzed during the current study are available from the corresponding author on reasonable request.

## Abbreviations

aOR: Adjusted odds ratio
CI: Confidence interval
COVID-19: Coronavirus disease-19
DOH: Department of Health
KAP: Knowledge, attitude, and practice
PCR: Polymerase chain reaction
SDO: School Division Office
WHO: World Health Organization
